# Supplementary material for: Impact of paleoclimate on present and future evolution of the Greenland Ice Sheet
Source: PLoS One. 2022 Jan 20;17(1):e0259816. doi: 10.1371/journal.pone.0259816 (PMC8776332; doi:10.1371/journal.pone.0259816)
Supplement: S1 File — (PDF) [file pone.0259816.s001.pdf]

Supporting Information for 'Impact of paleoclimate on present and future evolution of the Greenland Ice Sheet'

Hu Yang[1*], Uta Krebs-Kanzow[1], Thomas Kleiner[1], Dmitry Sidorenko[1], Christian Bernd Rodehacke[1,2], Xiaoxu Shi[1], Paul Gierz[1], Lu Niu[1], Evan J. Gowan[1,4], Sebastian Hinck[1], Xingxing Liu[1,3], Lennert B. Stap[1,5], Gerrit Lohmann[1]

1 Alfred Wegener Institute Helmholtz Centre for Polar and Marine Research, D-27570 Bremerhaven, Germany
2 Danish Meteorological Institute, DK-2100 Copenhagen Ø, Denmark
3 State Key Laboratory of Loess and Quaternary Geology, Institute of Earth Environment, Chinese Academy of Sciences, Xi'an, 710061, China
4 Department of Earth and Environmental Sciences, Kumamoto University, Kumamoto, Japan
5 Institute for Marine and Atmospheric research Utrecht, Utrecht University, 3584 CC Utrecht, Netherlands

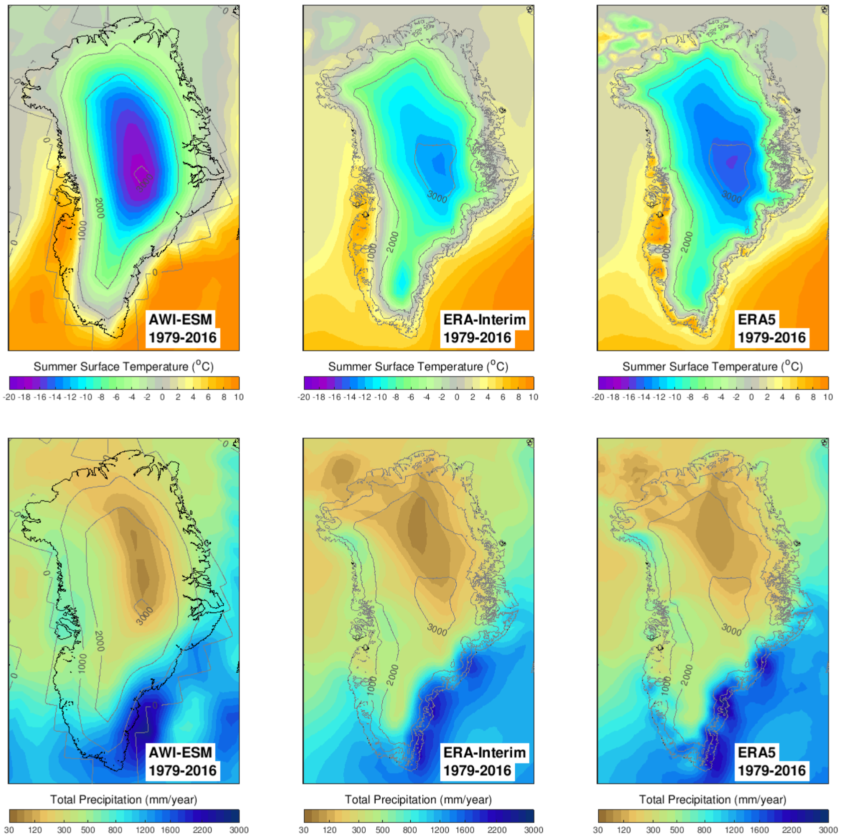

Fig. S1: **Comparison between our AWI-ESM model simulations and ob-servations (ERA-Interim (1) and ERA5 (2)), in terms of summer 2-m surface air temperature (top) and annual mean precipitation (bottom).**

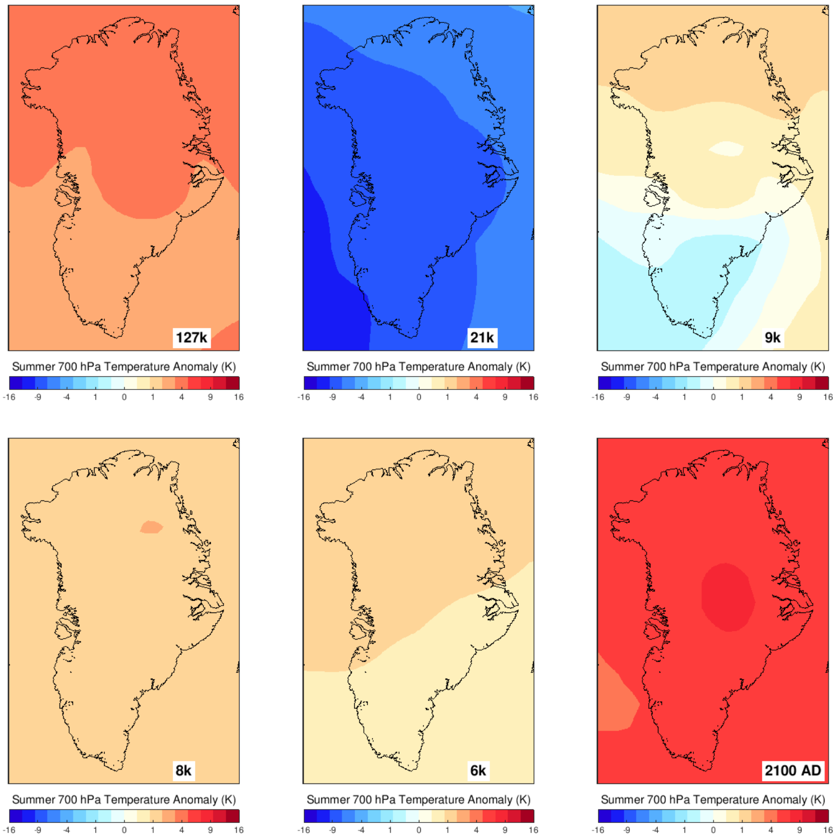

Fig. S2: **The AWI-ESM simulated summer (JJA) 700 hPa air temperature in different periods.** Plot as the anomaly in comparison with Pre-Industrial conditions. The contemporary coastline is highlighted in black line.

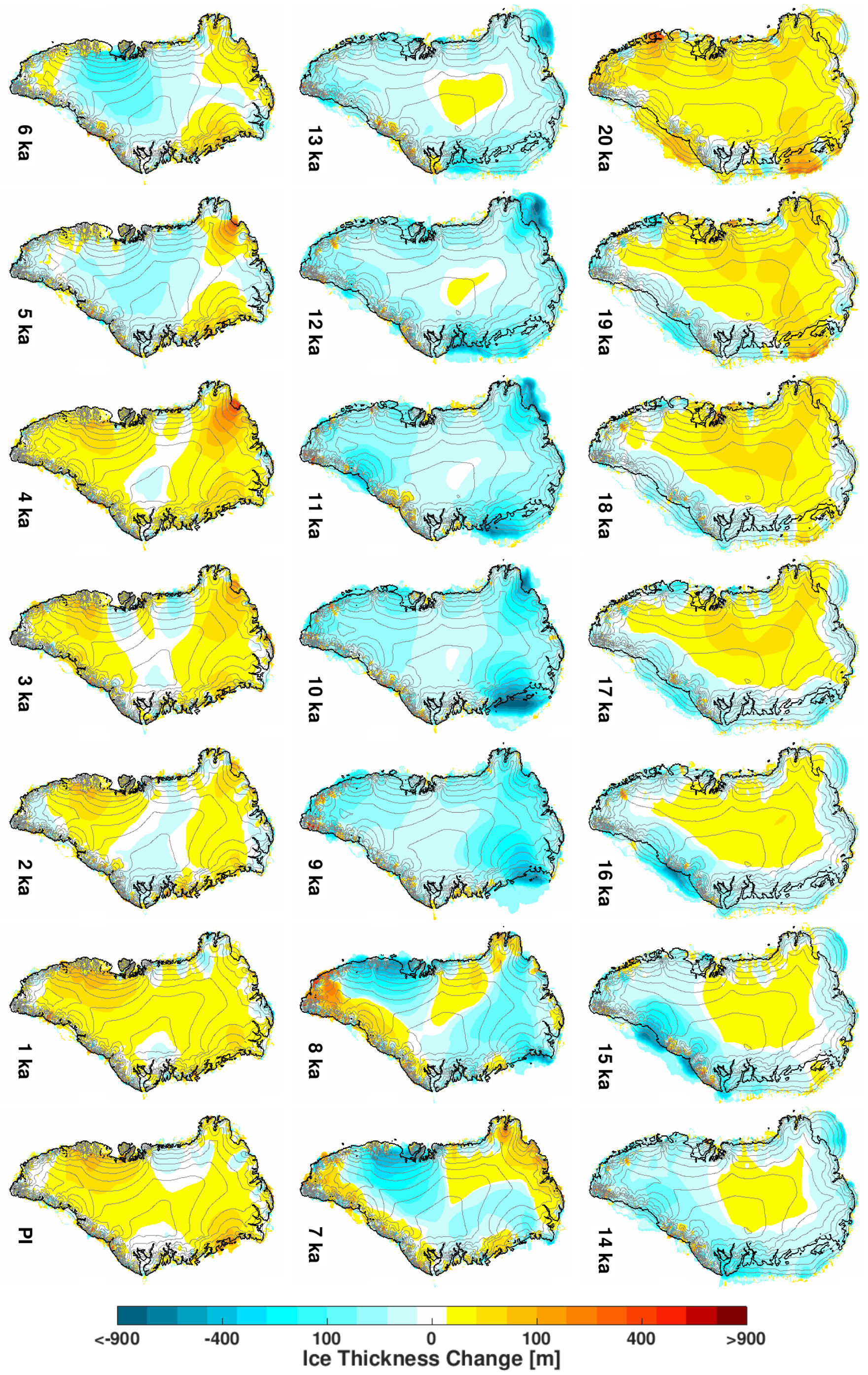

Fig. S3: **The simulated evolution of surface elevation of the GrIS from the Last Glacial Maximum (21 ka) to the Pre-Industrial era.** Shading presents the elevation change in 1000 year intervals. The grey contours give the surface elevation with 300 m intervals. The thick black line marks the coastline in the corresponding time. The results are based on one (the default one, see Table 1) of the ensemble simulations.

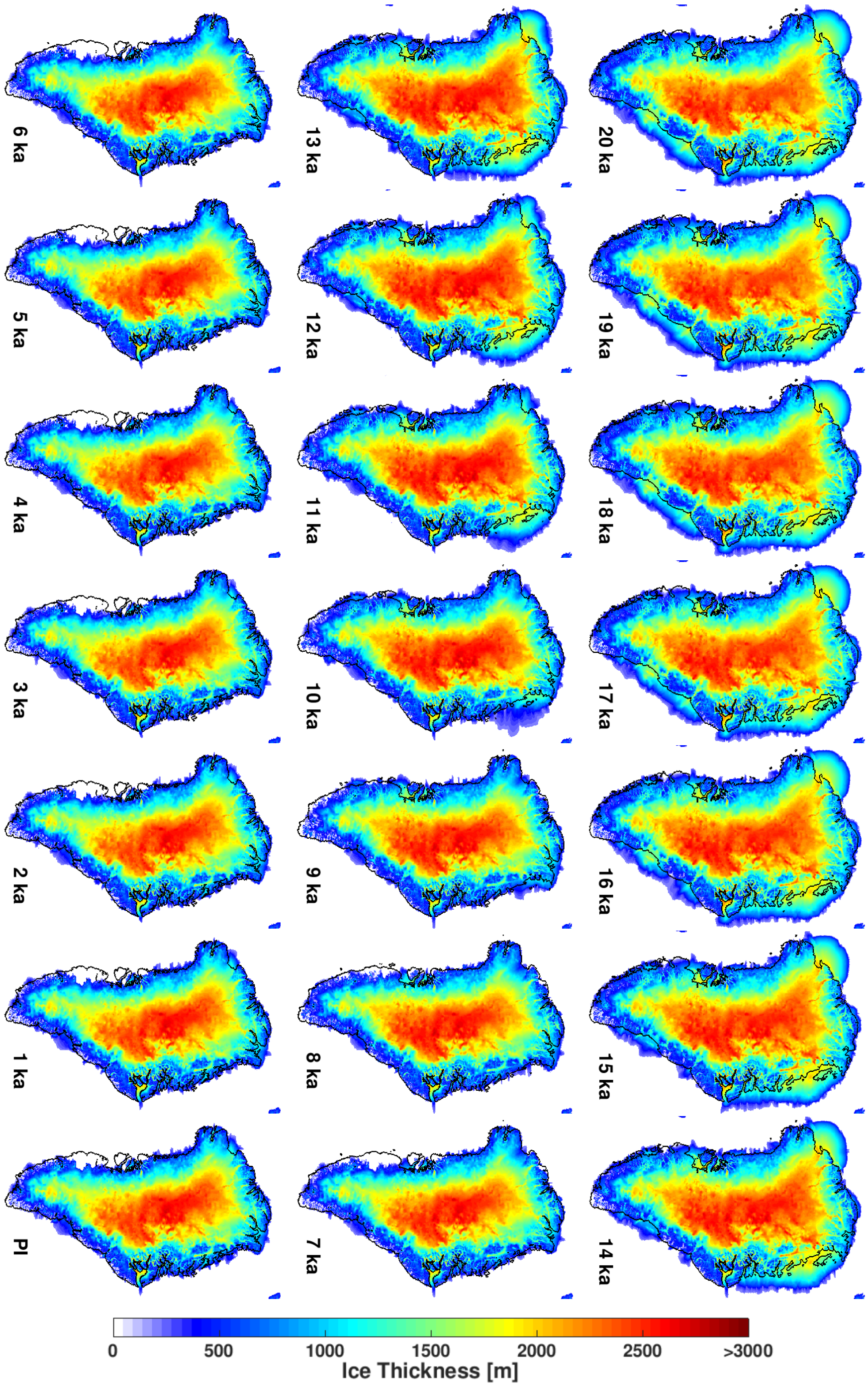

Fig. S4: **The simulated ice thickness evolution of the GrIS from the Last Glacial Maximum (21 ka) to the Pre-Industrial era.** The thick black line marks the coastline in the corresponding time.

References

1. Dee DP, Uppala S, Simmons A, Berrisford P, Poli P, Kobayashi S, et al. The ERA-Interim reanalysis: Configuration and performance of the data assimilation system. Quarterly Journal of the royal meteorological society. 2011;137(656):553–597.
2. Hersbach H, Bell B, Berrisford P, Hirahara S, Horányi A, Muñoz-Sabater J, et al. The ERA5 global reanalysis. Quarterly Journal of the Royal Meteorological Society. 2020;146(730):1999–2049.
