# Supplementary material for: Impact of paleoclimate on present and future evolution of the Greenland Ice Sheet
Source: PLoS One. 2022 Jan 20;17(1):e0259816. doi: 10.1371/journal.pone.0259816 (PMC8776332; doi:10.1371/journal.pone.0259816)
Supplement: Attachment — Submitted filename: PLos_ONE_Paper_Response Letter.docx [file pone.0259816.s002.docx]

Response Letter

Dear Editor,

Thank you for providing us the opportunity to submit a revision of our manuscript. We would also appreciate the time and effort that you and two reviewers dedicated to providing constructive comments.

Both reviewers have major concerns on the regional sea level forcing in our Greenland ice sheet simulations. The second reviewer also recommend us to carry out more ensemble simulations. As suggested, in the revision, we have included 7 ensemble simulations to perform the analyse. The sensitivity of regional sea level change to the model result is also tested.

Both reviewer have pointed out that the ocean temperature is likely an important forcing for the retreat of the GrIS during the last deglaciation, we agree on that. However, as limited by the current development of climate and ice sheet models, we are not able to provide new simulations which properly resolve this issue. Any simple treatment of ocean-ice shelf interaction would require heavy model tuning and introduce more uncertainties. Due to this flaw, and the concern on the reproduction of deglaciation climate, in the revision, we do not attempt to rigorously interpret our results before the Holocene. The simulation before the Holocene is rather treated as a long paleo spinup. We primarily focus on the impact of Holocene climate on the present and future evolution of the GrIS. Both paleo proxies and our simulations have indicated that the GrIS is mostly land-based after 11 ka before present, therefore, ocean forcing may play a minor role in the development of the GrIS after the Holocene.

In the previous version, our story line was not clear enough. There is an impression that we are reconstructing the past evolution of the GrIS using a numerical model. Therefore, the second reviewer has raised a lot comments asking us to validate our model result with observations from different perspectives, i.e., simulated pattern of temperature, precipitation, surface mass balance, dynamic ice loss, ice discharge, future sea level contribution, past sea level contribution, past climate anomalies. We are honest to say that our simulations are performed without any constraints from observations. Given the current limitations of ice sheet models, it is too optimistic to expect that our simulations could reproduce an ice sheet configuration that matches the observations, especially the magnitude of climate anomaly, ice sheet volumes and mass balance. However, the climate and ice sheet models are designed following physical laws, they can reproduce the general behaviour of the climate and the ice sheet, and therefore are useful for understanding the dynamics. Despite the reality that the simulated climate and ice sheet results are not suitable to be directly compared with observations, different simulations using the same model but with slight changes to parameters can be compared. For example, it is meaningful to compare the simulated evolution of the GrIS when spun-up from paleo state, and from a preindustrial state.

Therefore, the main focus of our study is to test how the paleo climate impacts the present and future evolution of the GrIS. We have created tests using several changes of the ice sheet model parameters to the paleo spin-up. We have reformulated the structure and discussion of the paper to fulfil this task. We would like to ask the second reviewer for understanding that part of his/her comments were not followed, as it would require substantial development work to implement them all.

Finally, in the revision, two new co-authors have been included, i.e., Evan J. Gowan and Lennert Stap. Evan is an expert on sea level and ice sheet reconstruction. He helped on the explanation of the regional sea level change around the GrIS. Lennert Stap is an ice sheet modeller. He helped on development of coupling of ice sheet model and solid earth model used in our response letter.

Please see below, in red, for a point-by-point response to the reviewers’ comments and concerns. All page numbers refer to the revised manuscript file with tracked changes.

Regards,

On behalf of all authors,

Hu Yang

Reviewer #1: Review of “Sea level response of Greenland Ice Sheet lags Climate Change by Millennia” (Yang et al.) by Tijn Berends

The Greenland ice-sheet is expected to contribute significantly to future sea-level rise. Due to its massive size, this ice sheet responds very slowly (on the human time scale) to changes in climate. This means that projections of future GrIS retreat cannot be feasibly based on observational evidence alone, and that ice-sheet models are needed to look into the future.

The authors present future projections of GrIS retreat under RCP8.5, performed with their ice-sheet model PISM, and forced with output from their own climate model, AWI-ESM. They pay particular attention to the different spin-up methods used to initialise their model, which is important since recent work shows that this can have a strong impact on the results. As the aptly chosen title of their paper reflects, their results demonstrate that the response of the GrIS to a change in climate, lags that climate change by several millennia – even longer than what’s commonly assumed (although, off the top of my head, I can’t name any studies that have actually made quantitative statements about this lag), and largely independent of the choice of spin-up method. This is relevant for both scientists and policymakers, as it illustrates that the commonly chosen “horizon” of the year 2100 is insufficient when talking about sea-level change.

A: Thank you for your brief summary. What you have written is just what we want to convey. We reformulate our title and structure to highlight that the aim of our study is to show that the GrIS response to climate change is slow, on the time scale of millennia. The slow response behaviour determines that the current evolution of the GrIS is a response of climate change both at present and also in the past. We also perform the actual impact of paleo climate on the present evolution of the GrIS.

I think this is an interesting study. However, before recommending publication, there are a few issues that I believe need to be addressed.

General comments

1) A large part of the manuscript is devoted to describing the two different initialization methods that were used for their two future projections, the “paleo spin-up” and the “equilibrium spin-up”. Two very extensive papers about this issue were recently published, one for Greenland (Goelzer et al., 2018) and one for Antarctica (Seroussi et al., 2019). I feel the discussion about the pros and cons of the different spin-up methods in the manuscript could be substantially extended. What general differences between paleo and equilibrium spin-ups did these other studies find, and how does that compare to your own results? What different explanations have been offered for these differences? (I suspect most of it relates to englacial temperature). I’d like to see a bit more context for this issue.

A: Previous studies have already discussed quite a lot on initialization strategies. We realize that our study is not really to show how to initialize the GrIS. The setup of our sensitivity simulation is rather to test how the past climate impact the present and future evolution of the GrIS. So, we changed our title and the story line.

Previously, our manuscript was separated into two parts, i.e., paleo evolution of the GrIS and initialization of the future projection of GrIS. In the revision, we connect those two parts logically, i.e., to understand how the GrIS evolved in the past and how the past climate and evolution of the GrIS impact the present and future evolution of the GrIS.

Coming back to the actual difference between the two initial conditions, we explored both the ice temperature and ice velocity (i.e., rate of dynamic ice loss). We find that the Pre-Industrial equilibrium spin-up tends to lead to a warmer ice temperature, which should contribute to a stronger ice loss under global warming. The paleo-spinup has memory of past glacial climate, this relatively cold temperature memory has been wiped out by the equilibrium spin-up (Fig. 7).

Also, the paleo simulation indicate that the GrIS was not in an equilibrium state approaching to the industrial era. The surface mass balance is higher than the rate of dynamic ice loss, contributing to increase the ice volume of the GrIS. However, in the equilibrium-spinup, the rate of dynamic ice loss is higher to balance the surface mass balance. This results in a stronger dynamic ice loss in the equilibrium spin-up than that of the paleo-spin-up. Stronger dynamic ice loss in the initial condition leads to more sea level contributions under global warming, if compared with the paleo-spin-up.

As suggested, the difference between those two initial conditions are discussed in detailed in the revision between line 437-448.

2) As an ice-sheet modeller myself, I found the ice-sheet model description a bit short. In particular, I’d like to know the following technical details:

a. Which version of the stress balance is solved in your version of PISM? From the context of the rest of the manuscript, I suspect it’s only the SIA?

A: PISM uses a model that combines the SIA and SSA. In areas where the ice sheet velocity is low, it solves for the SIA, while in ice shelf and fast flowing regions, it uses the SSA. We include such introduction between line 79-82.You can also find the realtive information in the manual of PISM as below:

At each time-step of a typical PISM run, the geometry, temperature, and basal strength of the ice sheet are included into stress (momentum) balance equations to determine the velocity of the flowing ice. The “full” stress balance equations for flowing ice form a non-Newtonian Stokes model. PISM does not attempt to solve the Stokes equations themselves, however. Instead it can numerically solve, in parallel, two different shallow approximations which are well-suited to ice sheet and ice shelf systems:

·	the non-sliding shallow ice approximation (SIA), also called the “lubrication approximation” , which describes ice as flowing by shear in planes parallel to the geoid, with a strong connection of the ice base to the bedrock, and

·	the shallow shelf approximation (SSA) , which describes a membrane-type flow of floating ice, or of grounded ice which is sliding over a weak base.

b. How do the “eigen calving and thickness calving parameterisations [that] are used to determine the calving-front dynamics” work? I am personally not familiar with these two parameterisations, and no references are provided where I can look them up.

A: The eigen calving and thickness calving parametrizations are used to determine the calving-front dynamics. Eigen calving is a simple formula for first-order kinematic contribution to iceberg calving, in which volume loss through calving at ice front is proportional to the determinant of the strain rate tensor, i.e. the product of its eigenvalue \cite{levermann2012kinematic}. When the thickness of ice shelf exceeds a certain thickness threshold, it is forced to be removed.

c. How does the calving rate / melt rate depend on ocean temperature? And why do you explicitly mention that it does, only to follow it up by stating that ocean temperature was kept constant during your simulations? Doesn’t that make the whole parameterisation obsolete?

A: This ocean model component implements boundary conditions at the ice/ocean interface that are constant both in space and time. The ice-ocean heat flux is determined following Beckmann and Goosse (2003) using a virtual temperature Tf, which is the freezing temperature of ocean water at the depth zb below the ice shelf.

Here So is the salinity of the ocean with a value of 35 psu. The transition from the base of the shelf with a salinity close to zero (Oerter et al., 1992) to the ocean water of full salinity occurs in the oceanic mixed layer which we cannot resolve in our approach. The resulting heat flux between ocean and ice, representing the melting effect of the ocean through both temperature and salt, is computed from

with ρo density of ocean water and cpo specific heat capacity of the ocean mixed layer. To is set to a constant value of -1.7 ◦C.

The above introduction can be found in the PISM manual and also shortly introduced in our revision between lines 79-110.

I think these are important details, because they all affect how your modelled ice sheet responds to prescribed changes in sea level. Looking at the top panel of Fig. 4, I see that, during the deglaciation, the changes in prescribed sea-level lag the changes in prescribed climate by several millennia (a number that sounds familiar!). I think it is important to see how strongly the modelled ice dynamics are influenced by the prescribed sea-level forcing, and therefore, how much of the observed lag in ice-sheet response is really just a more-or-less instantaneous response to the sea-level forcing, rather than a delayed response to the climate forcing. I’m not sure if it’s possible to really answer this question without having to do some extra modelling work, e.g. a simulation with constant sea-level forcing. Recent work has underlined the importance of changes in ocean temperature for the retreat rate of Jakobshavn Isbrae (e.g. Bondzio et al. [2018, Geophysical Research Letters] and Khazendar et al. [2019, Nature Geoscience]), and the importance of glacier retreat in general for the total mass balance of the GrIS (e.g. King et al. [2020, Communications Earth & Environment]). Based on that, I think it’s very important to pay more attention to the role of sea-level forcing in your model.

A: Indeed, we agree that the ocean temperature change during the last deglaciation would play an important role in the retreat of ice shelf of the GrIS. However, our climate simulations do not include an interactive ice shelf. The simulated ocean temperature may not able to reflect reality, especially during the summer when the sea ice retreats to the coast of the margins of the GrIS. There are some technical difficulties to include dynamic ocean temperature forcing. In the modern real world, research is still going on to understand how the deep ocean water exchange with the ocean water beneath the ice shelf. High resolution ocean and ice shelf model has developed to investigate this process. This study initiated prior to the release of this submodule, and implementing it would require us to restart all of the model runs.

Coming back to the sea level forcing, we perform several sensitivity simulations by including global mean sea level forcing with different magnitudes (Fig. R1). The results show that without global mean sea level drops, the GrIS can not grow enough big during the last glacial maximum. Global mean sea level drop does play a vital role for the development of the GrIS. We believed that part of the delayed in GrIS response to cold climate is due to the delayed sea level drop. However, sea level drop is also a fingerprint of growing ice sheet. It seems that there is positive feedback between sea level drop and growth of the Northern Hemisphere ice sheets. However, even if the global sea level fall was 70% of the observed value at the LGM, the Holocene configuration is similar to the 100% case (Fig. R1). A substantial change in configuration only happens if a very unrealistic sea level forcing is used.

Second reviewer has also raised up the similar comments on the ocean temperature and sea level forcing. We agree that there are large uncertainties in our model results before the Holocene, especially for the question of whether sea level, ocean temperature, or atmosphere temperature, is the main driver of deglaciation of the GrIS. Therefore, in the revision, we do not attempt to rigorously interpret our results before the Holocene. The simulation before Holocene is rather treated as a long paleo spinup.

Fig. R1. Evolution of ice volume of the GrIS under different sea level forcing. 0.0SL means that there is no global mean sea level change. While the other simulations are integrated with magnitude of global mean sea level forcing been scaled by different factors.

3) Some more details about the sea-level forcing:

a. How do you scale benthic d18O to global sea level?

A: The sea level reconstruction is from the PISM model developer. This curve has been used widely by the PISM user. According to the official website of PISM, it was reconstructed from J. Imbrie (1984). I was scaled linearly between lowest LGM value and present day values. However, sea level curve uncertainty due to different methods play a minor role in the results of the Holocene evolution of the GrIS.

Reference: J. Imbrie and eight others. The orbital theory of Pleistocene climate: Support from a revised chronology of the marine delta-O-18 record. In Milankovitch and Climate: Understanding the Response to Astronomical Forcing, pages 269–305. D. Reidel, 1984.

b. How do you scale global to local sea level?

A: The sea level forcing around the GrIS is the sum of the externally set global sea level curve, and the Earth deformation due to changes in the ice load from the Lingle-Clark model. This is a simple model that does not include other less significant components of the glacial isostatic adjustment signal (i.e. gravitational attraction, Earth rotational effects and effects from other ice sheets). Despite this, it should provide an adequate representation of local sea level change for the purposes of our experiments (check the Fig. R3).

The d18O signal you used is a combination of global sea level and deep-sea temperature. Several different studies (e.g. the inverse modelling work by Bas de Boer, and the data work by Jeremy Shakun) have shown that those do not always change in tandem. As your own results show, this is especially true during the deglaciation, which could affect the timing of your sea-level forcing, which might in turn affect you results.

A: Yes, the reconstruction of global mean sea level based on benthic d18O signal has uncertainty due to deep sea temperature changes. The sea level forcing we have used has a magnitude of around 130 m sea level drop during the last glacial maximum, which is derived based on a GIA model study (Lambeck et al, 2014). More recent studies show that this drop is likely too large by up to 20 m (Simms et al 2019, Gowan et al., 2021). Therefore, we perform an ensemble simulation to scale the sea level forcing with a factor of 0.9, which means the maximum sea level changes is around 130*0.9=117 m. The result show that magnitude of sea level change does have an impact on the magnitude of ice volume of the GrIS during the glacial period. However, the sea level forcing does not affect the main conclusion of our study, i.e., the several millennia lagging effect, the mid-Holocene minimum GrIS and background growing GrIS during the late-Holocene. The configuration of the ice sheet during the Holocene is not strongly dependent on global sea level so long as the magnitude is not reduced below a factor of 70% (Fig. R1).

Considering the uncertainties on the exact time and magnitude of sea level change during the last glacial maximum, we don’t emphasize an interpretation the evolution of the GrIS during last deglaciation in our revision. The simulation before the Holocene is treated as a long paleo spinup.

Why did you choose to use a d18O stack rather than an actual sea-level reconstruction?

A: Our simulation start from the Last Interglacial. Therefore, we need to use a sea level time series that covers the entire period of last glacial cycle. To our knowledge, the sea level reconstruction based on the d18O is the only continuous record of sea level that is available for the entire glacial cycle.

Then, there’s a difference between global and local sea-level. In the text you mention a sea-level low of -130 m at LGM, which is fine when you look at the global mean, but for the Greenland coast (so close to the Laurentide) that number would be significantly smaller. Again, this might or might not be important, depending on how strongly your ice dynamics are influenced by the prescribed sea-level forcing.

A: The second reviewer has raise the similar question. We have included a solid earth sub-model in our simulation, i.e., the Lingle-Clark model, which resolves the regional sea level change due to GIA. We agree that the regional sea level can be different if the gravitation of Laurentide and other ice sheets are taken into accounted.

The total effect of the global GIA signal in Greenland will be complicated. Indeed, the gravitational attraction of water towards the large ice masses will attract water towards Greenland, causing local sea level to be higher than the global average. The amount of gravitational attraction will depend on the distribution of ice. Working against this is the fact that Greenland will be in the forebulge region of the Laurentide ice sheet (and possibly the Eurasian ice sheets), which would make sea level lower than the global average. The magnitude of the forebulge will be dependent on assumptions of Earth rheology and the size and evolution of the ice sheets. Including these signals would add considerable complexity to our experiments. The largest component of the GIA will be related to global sea level fluctuations and local Earth deformation, so we consider other components to be small enough to not substantially alter our results.

To test how much the other ice sheet impact on the regional sea level around the GrIS, we have carried out a solid earth model simulation with Northern Hemisphere ice sheets forcing. The results indicate that the gravity induced by the Laurentide ice sheet has minor impact on the regional sea level around the GrIS (Fig. R2). The regional sea level change is primarily controlled by two factors, i.e., GIA due to local ice load and retreat, and the global mean sea level change. These two factors have both been considered in our GrIS simulations.

Please check also the response for the first comments from the second reviewer.

Figure R2. To isolate the impact of Laurentide and other Northern Hemisphere ice sheets on the regional sea level change around the Greenland, we perform a solid earth model simulation using VILMA. This simulation is initialized by the ETOPO1 modern topography at 125 ka before present, and forced with Northern Hemisphere ice sheet evolution (without changes in the Greenland Ice Sheet) taken from results of Niu et al. 2020. The above Figure A shows the ice thickness and regional sea level change during the LGM (21 ka) with respect to the present day (0 ka). Figure B gives solely the changes in the relative sea level. As shown, the regional sea level around the GrIS has not much difference from the global mean sea level changes, suggesting that the Laurentide ice sheet has little impact on the regional sea level change around the GrIS. The strongest relative sea level changes are found over the Laurentide ice sheets and the Eeurasian Ice Sheet, due to land deformation.

4) Two minor points:

a. Why did you choose the very old d18O stack from Imbrie et al. 1984, instead of the more commonly used LR04 stack, or the one by Ahn et al. (2017), to generate your sea-level forcing?

A: This sea level time series has been widely used also by several other ice sheet modellers, like Mackintosch et al. 2011, Golledge. Et al. 2012, Stuhne and Peltier 2015, Torsten et al. 2020. We realize that it is old. We would like to use the latest one in the future work. Anyway, the sea level forcing does not impact the main results of our study, as shown by our sensitivity simulation forced by different sea level forcing (Fig. R1)

b. Why did you choose the rather old Bamber et al. 2001 data, instead of the more recent Bedmachine data, to initialise your model?

A: Sorry, we made a wrong statement in the figure caption (in the main text it is correct). Actually, our initial condition is obtained and interpolated based on the ETOPO1 dataset. In the revision, this error has been corrected.

Specific comments

Since I expect the general comments above will require substantial changes to the text, I don’t believe it is useful to go into the small details right now.

A: We agree! Thanks a lot for your time and effort to provide constructive comments. I also learn a lot by exploring the answers for your questions.

Reviewer #2: Review of “Sea level response of Greenland Ice Sheet lags Climate Change by Millenia”

This manuscript describes the results of the modelled evolution of the Greenland Ice Sheet (GrIS) from the last glacial maximum (LGM) till 2100 using offline coupled ice sheet model PISM with AWI-ESM climate fields. This kind of simulations where ice sheet models are driven by ESM data are the future of reconstructing the past evolution of ice sheets and unravelling the drivers of their changes.

However, such experiments should be designed correctly and to my regret I have to conclude that authors have made several major errors. Therefore, this manuscript and the contributing scientific research need, in my view, major revisions before publication is possible.

The first, and most critical flaw is that the authors provide the global mean sea level curve to their PISM simulations. Honestly, I was amazed that researchers funded in a project called “Regional Sea Level Change and Society” are not aware of the fact this is erroneous. Due to the presences of the Laurentide Ice Sheet, the sea level around Greenland at LGM was about 50 to 80 meters higher than today and falling during the transition phase. This flaw invalidates all results shown in Figures 4 and 5, the analysis of Section 3 and hence the conclusions and title of the manuscript.

A: We noticed that the regional sea level variation is not written clearly in our manuscript, leading to a misunderstanding on our model setup. As the first reviewer has also had questions on the sea level forcing. We would explain this point in a more detailed way here.

Regional sea level changes around the GrIS are primarily driven by 3 factors.

First, the strongest impact, at least locally, is the deformation of the Earth induced by ice loading changes. This effect has been taken into accounted in our simulation, as we have included the solid earth deformation parameterization, i.e., the Lingle and Clark bed deformation model (see also Fig. R3).

Figure R3. PISM simulated Greenland relative sea level at 21 ka before present. The thick black line gives the ice margins of the GrIS. Indeed, there’s some region, which experience relative sea level drop from the 21 ka to PI. But these regions locate at the inland of the Greenland, the ice there has no direct contact with the ocean water. At the margins of the GrIS, most of the ice will experience a regional sea level rise, primarily due to the global mean sea level rise from 21 ka to PI.

Second, the global mean sea level change. During the last deglaciation, the global mean sea level rises about 100 meter within 10 kyr after the LGM, which is the second dominant factor driving the regional sea level change. This effect has also been taken into account in our simulation, as we have prescribed a global mean sea level curve to force the model.

Third, the gravitation induced by the Laurentide and other ice sheets. To get an impression of how much regional sea level change can the Laurentide ice sheet introduce to the GrIS, we perform a solid earth simulation using the VILMA 3D solid earth model. VILMA has considered all the factors that impact the regional sea level, including GIA, global mean sea level change, regional gravitation change due to land ice redistribution, and variation of the Earth’s rotation speed induced by redistribution of water mass. We run a VILMA simulation driven by evolution of the Northern Hemisphere Ice Sheet from the last interglacial to the pre-industrial. This ice sheet forcing is taken from Niu et al. 2020. To exclude the impact of evolution of the GrIS on the regional sea level change, we replace the GrIS by using a constant GrIS taken from ETOPO1 data. As shown in Figure R2, the regional sea level around the GrIS during 21 ka is around 120-140 m lower than present, not so much difference from the global mean sea level change.

We admit that we have not taken into account the gravitation effect, but this does not lead to fundamental wrong results. Regional sea level changes have been widely used to reconstruct the ice sheet. Considering that you have argued that part of the regional sea level over GrIS may 50-80 m higher than present during the last glacial maximum, this is primarily due to local ice loading. This kind of sea level change is slow as we can still observe it nowadays. It is mostly a response to the ice sheet changes. More importantly, we have considered such GIA effect in our model simulation (Fig. R3).

The second, and likely linked major flaw is the choice to use constant ocean temperatures (L216). In this study, rising sea level pushed the GrIS on land. However, it is far more likely that rising ocean temperatures shredded the LGM ice shelves of the GrIS, despite the falling regional sea level. I can imagine that the “sea level error” clouded this oversight during the tuning phase of this project. However, in the revised PISM simulations, this needs to be corrected as well.

A: Thank you for pointing out a critical question. Indeed, we expect that the ocean temperature could play a major role for the retreat of ice that is grounded on the ocean floor, especially during the last deglaciation. However, at the current stage, we are not able to perform such a simulation, not only because of time, but also due to technical reasons.

The ice shelf interaction with ocean is complicated. As our climate model has not included an interactive ice sheet, the simulated ocean temperature close to the ice edge may not be realistic, especially during summer time when the sea ice has retreated to the position close to the continental shelf of the Greenland. Also, how the ocean water circulates beneath the ice shelf is not well known even in modern ice shelves. AWI does have simulations with interactive ice shelf with ocean, but that simulation is based on very high resolution ocean standalone model. Implementation of such a model is beyond the scope of our study, as the computational demands are too great to run on glacial time scales.

The raising sea level in our model plays a role to cut the ice shelf, as we have used thickness calving parametrizations, in which when the thickness of ice shelf is over certain threshold (i.e., 250 m), it is forced to be calved. During deglaciation, raising global mean sea level contribute to cut the ice shelf and therefore, contribute to the retreat of the GrIS. The raising sea level can somehow mimic the retreat the ice shelf due to ocean forcing, no matter it is forced by temperature or sea level rise.

In addition to this, there is no evidence of an extensive Antarctic-like ice shelf in the LGM Greenland Ice Sheet (Jennings et al., 2018). The Greenland Ice Sheet likely remained grounded to the continental shelf with only a limited ice shelf, so sub-shelf processes were not likely to have played as large of a role in the ice sheet dynamics as in the modern Antarctic Ice Sheets. Properly characterizing the marginal conditions of the LGM Greenland Ice Sheet would likely demand the development of a completely new model of ice-ocean interaction. This is beyond the scope of this study, as we are interested in the predominantly land-terminating Holocene evolution of the ice sheet to present day.

Considering this flaw, with ocean temperature forcing not properly parameterized, we now consider the simulation before the Holocene as a paleo-spinup for the Holocene results. We now primarily focus on our interpretation on the model results starting from the Holocene Thermal Maximum. Based on geologic evidence of ice margins, the GrIS was mostly land-based after 11 ka. So, we expect that our simulations during the Holocene would be more realistic, despite the lack of ocean temperature forcing.

The third shortcoming is the use of the Positive Degree Day (PDD) method to determine ice ablation. It has been demonstrated numerous times that PDD fails to include the impact of varying summer insolation over the course of glacial cycles. The authors should use a temperature-insolation model (e.g., de Boer et al, 2013, doi: 10.1007/s00382-012-1562-2, Eq. 30) which is physically more correct even if a constant albedo is used. This will increase melt during the retreat phase of the GrIS.

A: This is a very valid point. We agree that the response to Holocene climate change is likely underestimated due to the neglected effect of insolation and we expect that the influence of the climate history will be even bigger than what is seen in our experiment. We added the following lines to our discussion:

*"Furthermore we can assume that the response of the GrIS to Holocene climate change is additionally underestimated in our experiments, as the here applied PDD method does not account for the variations in insolation on orbital time scales. During the last interglacial the change in insolation substantially contributed to surface melt on the GrIS \citep{vandeberg2011, plach2018}. For the Mid Holocene, \citet{UKK2021} estimate that PPD schemes will underestimate surface melt by approximately 10\% due to the neglected effect of insolation.“*

Fourthly, I was puzzled by the fact that the authors present only one realization of PISM for the period between the LGM and 1850. If model simulations are very expensive – several months on a supercomputer – that is a sacrifice one needs to do. However, PISM at 5 km is likely expensive but I don’t see the necessity to present only one realization. The authors should demonstrate that different choices with respect to model tuning and experiment design would not lead to different conclusions. For example, the glacial index method is used prior the LGM, but not for the long timespan between 21 and 9 ky BP, during which the temperature neither changed linearly. Would the evolution of the GrIS in PISM be fundamentally different if the glacial index method would be used too? This discussion should be added to the manuscript. Otherwise, if PISM is indeed too expensive for multiple realizations, the authors should explicitly state this.

A: Thanks for your suggestion. In the revision, we have included 7 ensemble simulations by changing the model parameters and the magnitude of the sea level forcing. Different ensemble members do produce different results on the magnitude of the GrIS ice volume and exact time of the peak size of the GrIS. However, they do show a consistent pattern of thousand years delay response of ice volume to climate change. Also, all the simulation show a minimum GrIS during the mid-Holocene.

We believe that prescribing the climate forcing with different strategies could lead to different climate and therefore different evolution of the GrIS. We recognize that the simulation before the Holocene has several uncertainties regarding the sea level forcing, ocean temperature forcing, and also the strategies to generate transient climate forcing. Therefore, in the revision, we do not interpret our results before the Holocene seriously. They are used as a long paleo-spinup for the initialization of the Holocene simulation.

Finally, it is a pity no AWI-ESM simulation is carried out for an intermediate slice between 21 and 9 ky BP. I know, it hard to pick a point due to high variability between 15 and 11.5 ky BP – caused by processes possibly not resolved by AWI-ESM –, and it in neither proposed for PMIP4, nonetheless such an additional time slice is worth considering.

A: Thanks for your understanding. The last deglaciation is difficult to simulate as it is a transient period with changing GHG, earth orbit, topography, sea level, land-sea mask and ice sheet extend and height. The ocean temperature was keep raising during this period. So, there is no actual equilibrium climate. Realistic simulation should start at the last glacial maximum. Considering that AWI-ESM has relatively high resolution, it would take 1-2 year to complete a deglaciation simulation, if everything works smoothly. Currently, AWI is developing an earth system model to include the adaptive land-sea mask, interactive ice sheet, solid earth, hydrological discharge model, which will hopefully help on such a deglaciation simulation.

Concluding, in order to make this study contributing to the advance of scientific knowledge, the author should improve their experiments by correcting the three experiment design errors, of which the first two are the most critical. I’m sure that paleo ice dynamics groups running sea level models are willing to share their estimates of the regional sea level history around Greenland. Oceanic temperatures can be extracted from the AWI-ESM. A subsequently revised manuscript should of course address the other comments which are given below.

A: This comment has been addressed by answering the previous comments.

However, when it is really impossible to redo the PISM simulations because, for example, the project has already ended, the title, abstract and manuscript but be plainly clear that the simulation considers a hypothetical situation that the GrIS faced a raising sea level during the transition from glacial to interglacial, and that the results have little predictive value for the real behaviour of the GrIS. I leave it to the editor to decide whether PLOS ONE is an appropriate platform for such an academic study.

A: As addressed above, there is a misunderstanding on the global mean sea level forcing. Without dropping the global mean sea level, the GrIS can not grow big enough during the last glacial maximum. In the revision, we do not sell our simulation result before the Holocene. We pay more attention on the impact of Holocene climate on the present evolution of the GrIS.

Other major concerns:

Evaluation of model results against observations for paleoclimate studies is indeed not easy, however, this manuscript provides very little evaluation of the model results, hence the modelled temperatures (Fig. 2), precipitation (Fig. 3), contemporary ice sheet mass fluxes (Fig. 4) and mass budget (Fig. 6) and possible future mass loss. This needs to be improved. More detailed comments are given below.

A: All the results given in our study are based on model simulation. We have not constrained the climate and ice sheet with any observation. Therefore, the scope of our study is not to reproduce the past evolution of the GrIS, but rather a simulation to mimic the dynamic of the GrIS.

Despite the fact that we are not able to reproduce exact magnitude of climate anomaly, ice volume, and ice geometry, the climate model and ice sheet model are designed based on physical laws. Therefore, the model results using different parameters can be compared to each other. Using states of the pre-industrial climate and GrIS as a reference, our model can reproduce a relatively warm Holocene climate and relatively cold last glacial maximum climate. Also, our model can simulate a bigger GrIS during last deglaciation and smaller GrIS during the mid-Holocene. These are in agreement with reconstruction.

However, as our model does not assimilate observations, it is too ambitious to really compare it with the observation, especially trying to fit all the magnitudes. Anyway, we have included the discussion on the uncertainty of the simulated climate and ice sheet volumes.

It might be an overlook in how the wording of a results pans out, but (L286-288) “Our results indicate that the …” is too much honour for a single realisation of a single ice sheet model. The results presented here align, affirm or question our current understanding of the transient behaviour of the GrIS. Even though at many other places in the manuscript do reflect that the model results are not necessary a good representation of the true past GrIS history, using “indicating” is not appropriate in here. In the revised manuscript, choose the wording of the results carefully.

A: Thanks for your correction. In the revision, we have included 7 ensemble simulations.

Indeed, we don’t consider our simulation as a reconstruction of past evolution of the GrIS. We position our study as a sensitivity test on how the past climate impact the present and future evolution of the GrIS. In the revision, we use more imply, hint, likely.

In general, the discussion misses, in my opinion, the point it could make. What other papers already have demonstrated is that the ice sheet model initialisation matters, that the Holocene was warmer than the preindustrial climate and that following the RCP8.5 scenario will onset deglaciation of Greenland. It makes little sense to focus solely on reaffirming knowledge. Instead, focus on the novel results this study provide. Transient simulations of the GrIS evolution driven by ESM data are sparse of non-existent. How certain is it in this study that 6-5 ky BP the GrIS was smaller than today and what kind of uncertainty range do you give to such estimate? At some points in the manuscript (e.g. L535), it is implied that many more PISM realizations are made in preparation to this manuscript. Discuss the sensitivity of the results to these choices in more detail.

A: Thank you very much for your suggestion on the novelty of our study. Our simulation has not been constrained by observations, therefore, the simulation should not be considered as a reconstruction of GrIS. We use a consistent climate forcing which generated by the climate model to simulate the evolution of the GrIS both in the past and in the future. Previous studies either focus on the past or the future. We are able to explore the impact of past climate on the present and future evolution of GrIS. Therefore, based on this story line, we rewrote our title and many parts of the manuscript. Our simulation can qualitatively test the impact of paleo-climate on the present and future evolutions of the GrIS.

Other remarks:

L167: Modelled summer near-surface temperatures over the central part of the ice sheet depend, besides regional tropospheric warming and topographic changes, strongly on the induced albedo decay by snow metamorphism. That is hard to model right, even for more dedicated models. It would be insightful to provide temperatures at a tropospheric level above the ice sheet as well (500 or 700 hPa) as well in supplementary materials.

Even though this paper is not focussing on model evaluation and observational data is sparse, it is not enough to only present and analyse the modelled surface temperatures. The authors must give an assessment of the quality and uncertainty range of the modelled temperatures, either by comparing to PMIP4 simulations of other groups and/or by comparing to observations. And if this is done extensively in other papers within the project, this evaluation or comparison results should be summarized here.

Figure R4. Summer air temperature anomaly at the 700 hPa level during different climate intervals.

Figure R5. Comparsion between our model simulated summer temperature and annual mean precipitation with that from the ERA5 and ERA-interim reanalysis. Our model can well reproduce the pattern of temperature and precipitation. Also, we can get the order of magnitude correctly.

A: As suggested, in the revision, we compare our model results with other reconstructions during the Holocene. Paleo reconstruction suggest that the Greenland peak temperature was about 2-7 degrees warmer than pre-industrial. However, our model only simulates a warmth of around 1 degree. Part of the reason may because we did not use an interactive ice sheets. We do compare and discuss this issue in our revision between line 476-500.

L190: Ref 46 is extremely outdated. Take an RCM based estimated precipitation field published in the past few years (e.g. [www.the-cryosphere.net/6/1275/2012/](http://www.the-cryosphere.net/6/1275/2012/) or <https://doi.org/10.5194/tc-2019-321>) as approximation of the real precipitation across Greenland.

A: Thanks. The new observational data is used.

L190: Similar as for temperature, add an assessment of the quality of the modelled precipitation by comparing to other GCM outcomes and accumulation records of ice cores.

A: As mentioned above, our study is not to reproduce the exact magnitude of climate and ice volume. Any such work would need enormous work on the model tuning. We consider our study as a sensitivity test on how the paleo climate impact the present and future evolution of the GrIS.

L210: Present day mass fluxes (1070 Gt yr-1 precipitation, 500 Gt yr-1 runoff, 570 Gt yr-1 ice discharge) are well above observed present day/preindustrial values – their values are about and 750, 300 and 500 Gt yr-1, respectively). In particularly precipitation and runoff are higher than observed. Please comment on the cause of these differences (the high runoff may be a choice to make the behaviour of the ice sheet match reality) and the impact on the modelled response presented in this manuscript.

A: We notice that there is difference between our model results and the observation taken from the last few decades. Several reasons can lead these discrepancies. Both our climate and ice model are not constrained by any observations of sea surface temperature, sea ice coverage, ice extend, surface ice velocity and ice temperature. It is reasonable to have difference simulated values. Despite many efforts to improve the ice sheet models, ice sheet models are still in their infant phase. It is too optimistic to expect that it can reproduce the observed values, especially for a simulation not constrained by observations. For example, the surface mass balance is influenced by the surface air temperature, ice elevation, precipitation, ice extend. To get this value close to observation, the above parameters should all be close to the observation. These requirements are far too strict for our simulations starting from the last interglacial and integrated based on simple assumption (i.e., glacial index method). We would ask the reviewer for understanding the difficulty in achieving complete realism. We would say, to get within an order of magnitude correct is already reasonable at the current stage. Therefore, our simulations are not ambitiously focusing on reproducing the observation. We position ourselves as a sensitivity test on how the past climate impact the present evolution of the GrIS. Including the discussion of comparison between observation and model value could mislead the main idea of this study. Numerical models are used to understand the physics and the sensitivity of some key processes, because these model results can be compared with themselves, not observations.

Figure 6 / Section 4: It is too bad that in Figure 6 and section 4 no comparison is made to observed mass loss of the GrIS, e.g. as presented in Shepard et al, 2019 (<https://doi.org/10.1038/s41586-019-1855-2>, fig. 3). This could be graphically done by showing the mass change since (e.g.) 2000 in Figure 6 (besides temperature).

Furthermore, 4.5 cm SLE mass loss from the GrIS under a RCP8.5 scenario is at the very low end of projections. Discuss why and the impact of this deviation on the interpretation of the model results.

A: We recognize that the magnitude of the temperature and ice volume can be different if the model parameterization changed. Therefore, we note in our discussion that the magnitude of ice volume should not be interpreted seriously. However, model simulation can compare with itself. Our statement of underestimate of sea level contribution is based on the comparison to the equilibrium spin-up.

We don’t think the current model development can be used to predict the future sea level rise correctly, because many of the physical processes, including the diurnal temperature variation, solar insolation change, subglacial hydrology, and albedo decay by snow metamorphism and melt ponds are not included. Therefore, we don’t claim that we are here to reproduce the past or future evolution of GrIS.

I’m a bit puzzled by Section 5. L312-315 state that the equilibrium-spinup simulation immediately starts loosing mass since 1990 (which agrees with observations) and might lose 7 cm SLE by 2100 under RCP8.5 (which also agrees more with other estimates). Nonetheless, the authors raise the suggestion that the paleo-initialisation is better despite the worse results. I agree that a paleo spinup is better than an equilibrium spinup. Nonetheless, the authors must acknowledge somehow this physical argument is not supported by the results presented now. Furthermore, the observation that the initialisation matters is already discussed in great lengths in several papers.

A: We are sorry that we made a mistake on the text. From our figure, it shows that the equilibrium spinup show mass loss starting around the 1890s, not the 1990s, nearly one century earlier than the paleo-spinup which started around the 1970s. The reason for this discrepancy has been discussed extensively in the revision. Again, we have no confidence to say which value is close to reality. But we have confidence to say without including the paleo-climate information, the sea level contribution will be higher than the simulation starting with the equilibrium spinup.

The model simulation and reality like two different worlds. Both world have its own benchmark (i.e., the pre-industrial condition) and can be compared with itself, but not each other.

L328-L337: please add the estimations of Simpson and Lecavalier to Figure 4b and discuss the agreement and differences with the results presented here in Section 3. I’m aware that the 4.6 additional SLE in Simpson is much more than presented here, leading to a cropped graph is this single point is added. Nonetheless, the timing of the maximum ice volume can be added easily.

A: We don’t consider our study as a reconstruction of GrIS, as we did not constrain the model with any observations. Our study is a sensitivity test on how the past climate impact the present and future evolution of the GrIS.

L333: “should not be interpreted seriously”. Rephrase, e.g. “It is worth noting that our simulated GrIS ice volume magnitudes depend (strongly/somewhat/slightly) on the choice of … anomaly. [And add a figure where these various realizations are displayed].

A: We have given an ensemble simulations with 7 members. The text has been reformulated.

L338-356: This paragraph is not very specific whether observations are or are not in line with the model results. Are proxies of Holocene temperatures in line with the modelled temperatures? Are these reconstructions of the GrIS (citations 7, 8 and 52) in line with the modelled evolution, or is your realisation retreated at other spots or thinner or thicker? I’m missing making a real connection with preceding work.

A: We have checked the reconstruction of past climate and our model simulation. It seems that our climate model has underestimate the temperature signal, hence the magnitude of ice sheet volume change. This has been discussed in the revised discussion.

L357-368: Again, the first part of this paragraph is an evaluation of the model results without comparing these other studies with the results presented here. Move the comparison part to section 4 and focus on the interpretation of the results. Finally, a proper conclusions section, hence in which is summarized what we have learned from this exercise, is missing.

A: We have included a new section of conclusions in the revision.

Figure 4: “Residual mass balance” phrases the view of the modeller as being the mass loss of the model that cannot be accounted to explicitly specified processes, hence SMB. However, phrase it as the processes it represents, thus presumably marine ice loss, combining basal melt of ice shelves and mass loss through calving. I presume basal melt of grounded ice is negligible. Furthermore, make clear in the caption (and text) if the curve shown is including or excluding ice shelves and if it is mass above flotation or total ice sheet volume. It is worth considering to show both (grounded ice mass above flotation, total ice mass including ice shelves)

A: In PISM, the total ice mass balance is determined by, surface mass balance, dynamic ice loss (i.e., ice discharge+calving), grounded basal ice flux, sub-shelf ice flux. The later two contributors are very small, almost two order of magnitude smaller than the surface mass balance and dynamic ice loss. The mass balance of GrIS is primarily controlled by the surface mass balance and dynamic ice loss. In the revision, we have specific it more clearly.

References:

Lambeck, K., Rouby, H., Purcell, A., Sun, Y. and Sambridge, M., 2014. Sea level and global ice volumes from the Last Glacial Maximum to the Holocene. Proceedings of the National Academy of Sciences, 111(43), pp.15296-15303.

Simms, A.R., Lisiecki, L., Gebbie, G., Whitehouse, P.L. and Clark, J.F., 2019. Balancing the last glacial maximum (LGM) sea-level budget. Quaternary Science Reviews, 205, pp.143-153.

Jennings, A.E., Andrews, J.T., Cofaigh, C.Ó., St-Onge, G., Belt, S., Cabedo-Sanz, P., Pearce, C., Hillaire-Marcel, C. and Campbell, D.C., 2018. Baffin Bay paleoenvironments in the LGM and HS1: Resolving the ice-shelf question. Marine Geology, 402, pp.5-16.

Krebs-Kanzow, U., Gierz, P. and Lohmann, G., 2018. Brief communication: An ice surface melt scheme including the diurnal cycle of solar radiation. The Cryosphere, 12(12), pp.3923-3930.
