# Supplementary material for: Impact of paleoclimate on present and future evolution of the Greenland Ice Sheet
Source: PLoS One. 2022 Jan 20;17(1):e0259816. doi: 10.1371/journal.pone.0259816 (PMC8776332; doi:10.1371/journal.pone.0259816)
Supplement: Attachment — Submitted filename: Response_Letter.docx [file pone.0259816.s003.docx]

Response Letter

Dear Editor,

Thank you for your effort to deal with our paper. We would also like express our appreciation for the two reviewers for their time and professional knowledge for improving the manuscript.

We have addressed all concerns and made for clarity a revised version where all the critics of the referees are taken into account. Enclosed are the point by point answers to the reviewers. The black colour is used for the comments from reviewers, red for our answer, and green for the text in the revised manuscript. All the line numbers are referred to the tracked change version of the revision.

On behalf of all co-authors,

Hu Yang

Reviewer #1: In this work the authors present simulations of the Greenland Ice Sheet (GrIS) evolution from the last deglaciation to 2100 performed by means of an ice-sheet – earth system model framework coupled in an offline mode. The authors suggest that, due to the time lag in the ice sheet response to the climate forcing, the 20th century retreat still shows some reminiscence of the late Holocene cooling. This also applies to future mass-loss projections, meaning that a paleoclimate spinup is required not to overestimate the future ice-sheet contribution to sea-level (SL) rise.

The experiment conducted here and the results achieved are certainly interesting. However, I believe there are still one major concern that the authors did not completely solve after the first round of review.

As other reviewers already pointed out, the oceanic forcing implemented in this work is inappropriate, as considering constant oceanic conditions since the last deglaciation is really unrealistic. In this experimental setup, the coastal retreat is only ensured by sea level changes through the flotation criterion, while any ocean-induced melting at the ice-water interface is neglected. The lack of evidence suggesting an ice shelf as large as those of Antarctica, as the author state, does not guarantee that sub-shelf melting played a minor role in the retreat. In fact, it’s plenty of work showing 1. the crucial role of the ocean in the past retreat since the last deglaciation (e.g. Jennings et al., 2017, Syring et al., 2020), but also 2. the importance of modelling oceanic changes to well simulate the past (e.g. Bradley et al., 2018, Tabone et al., 2018), 3. the current state (e.g. Rignot et al., 2016, Cowton et al., 2018, Morlighem et al., 2016) and 4. the future (Slater et al., 2020, Goelzer et al., 2020; Choi et al., 2017) of the Greenland ice sheet. Experiments such as ISMIP6 (Goelzer et al., 2020), yet with simple oceanic melt parametrisations, take into account oceanic changes in future projections, although the current percentage of the GrIS in contact with the ocean will likely decrease more. Since this study focuses on past and future retreat simulations, I would strongly suggest to consider at least a time-dependent oceanic temperature to capture the oceanic variability at regional spatial scales. This would mean repeating the experiments, but since the authors have already expressed their unwillingness to do it, this issue should be at least discussed thoroughly as it’s a critical flaw of the research. I don’t think this is properly done in the current version of the manuscript.

A: As suggested, all our simulations were repeated with a time-dependent ocean temperature forcing. The related figures have all been updated by the new simulation results. We extracted the coastal ocean temperature within 100 meters to derives a mean ocean temperature based on NGRIP ice core. Our model output give a mean ocean temperature of -1.43 at LGM and -0.19 at Pre-industrial era. Our simulation show that including ocean temperature produce a smaller GrIS during the last interglacial period and a slightly bigger GrIS during the last glacial period (Fig. R1), in respect to the simulations without a temporal ocean temperature forcing. However, there is no significant difference on the general evolution of the GrIS. The possible reason may because that our atmospheric and oceanic forcing is extracted from the same source, i.e., AWI-ESM. Therefore, ocean temperature signal is also included in the spatial air temperature field. Raising ocean temperature, in part, been reflected by the raising coastal atmospheric temperature.

Fig R1. A: GrIS simulation with time-dependent ocean temperature forcing (blue line) and the one without a time-dependent ocean temperature (red line). B: The difference between the two experiments. The results are based on the ensemble mean of the 7 ensemble members.

Fig. 4a of the revised manuscript. Evolution of the climate forcing applied in the GrIS simulations.

Another (minor) concern is related to the climate forcing strategy applied to the last 21ka. Why not using the same climatic index applied between 128 ka to 21 ka? In this way, abrupt events such as the Bolling Allerod could be perceived by the climatic forcing and reflected in the ice-sheet retreat. Are the authors sure that a linear interpolation until the HTM does not affect the climatic background that the GrIS would be still subjected to? I just found strange that such attention to the millennial variability is reserved for the spinup and then neglected for the most interesting part of the experiment.

A: Glacial index method is widely used in the ice sheet modelling. However, this method has its shortcoming, especially for reproducing the seasonality of the past climate change.

Firstly, the past climate change, especially the last deglaciation, is largely driven by redistribution of solar radiation at different seasons. Deglaciation warming occurs primarily during the summer time, due to Earth’s obliquity and precession change. However, the millennial variability during the last deglaciation is primarily a result of AMOC fluctuations (McManus et al. 2004: Collapse and rapid resumption of Atlantic meridional circulation linked to deglacial climate changes), which features mainly winter temperature change (Buizert et al., 2018). Therefore, using NGRIP driven temperature signal can overestimate the summer warmth during the Bolling Allerod, which ultimately may not be a good way to reproduce the real climate history.

Secondly, glacial index method is suitable to reproduce the climate between two significant different background climate states, like the climate between the last glacial maximum and the last interglacial, as most of the glacial index variabilities are within both climate states. However, when applying this method for some climate state which are not significantly different from each other, like the Holocene and pre-industrial era, the ice core variability sometimes can exceed the range between these two climate states, artificially producing unrealistic large-scale climate anomaly.

Thirdly, there are a lot of different ice cores taken from the Greenland ice sheet. They did not show identical temporal evolution, depending on the regional climate signal. Glacial index method can amplify the regional climate anomaly into large-scale climate anomaly.

In the revision, we explain why we use a linear transient climate after 21 ka (lines between 150-159), and have also discuss the shortcoming of this study (lines between 402-409), i.e., used a simplified method to derive the past climate change. This should be improved in the future by more realistic fully coupled earth system model or other strategies.

List of minor changes (only those I picked, please read the manuscript carefully to avoid typos/english mistakes):

A: Thanks for your carefully read. We have done this in the new revision.

Age: I’d rather see “26 ka BP” or “26 ka ago” or “-26 ka” instead of “26 ka”. Please, check the journal policy on that.

A: As there are a lot ‘kiloyear before present’ in the paper, we use “ka” as an abbreviation. This abbreviation is introduced the first time when they presents in the paper. I also checked other paleoclimate paper, some of them used the same way.

Line 36: “Considering that the available studies focus on the evolution of the GrIS either in
the past or into the future, the impact of past climate on present and future evolution
of the GrIS is not well known”. This might be true, but the authors are completely bypassing the fact that most of work of stand-alone ice sheet models cited here does proper glacial spinups to perform future projections (e.g. Applegate et al., 2012, Yan et al., 2013, Calov et al., 2018, ... and also Fuerst et al., 2015, Rueckamp et al. 2019, not cited) thus there is a certain “paleoclimatic background” in those future simulations. Please, add a small paragraph on this, especially since it’s related to your conclusions. Moreover, I found the introduction pretty short for the work you are presenting. Please, consider adding some relevant work focusing on the past (e.g. Buizert et al., 2018, Tabone et al., 2018) and the future (e.g. Fuerst et al., 2015, Rueckamp et al. 2019) evolution of the ice sheet.

A: All these referenced are cited and we have added one paragraph here.

‘ Besides the studies on the past evolution of the GrIS, a great effort has been carried out to predict the future evolution of the GrIS [22-29]. Depending on the greenhouse gases emissions scenarios and numerical model strategies, studies have projected that the GrIS may contribute to 2 cm to 33 cm of sea level rise by the year 2100. By integrating the GrIS model over the next millennium, Aschwanden et al. [30] illustrates that the GrIS may melt away under the strongest warming scenario. In the exercise of projecting the future evolution of the GrIS, many studies find that different initialisation methods affect the future projections of the GrIS [23, 24, 31, 32]. Aschwanden et al. [31] found that the short-term response of ice sheet models is strongly influenced by the initial state. Therefore, it is essential for numerical simulations to provide an initial model state that is consistent with the climate forcing [24]. Previously, two major initialisation strategies were widely used, i.e., paleo-spinups and equilibrium-spinups. The paleo-spinup strategy uses the evolution of past climate to force the ice sheet model to present, while the equilibrium-spinup applies observed climate from the present (or pre-industrial) to force the model into an equilibrium state. Yan et al. [23] noticed that simulations with paleo-spinup produce results closer to observations than those with equilibrium-spinup.’

Section 1.3: I suggest to precisely describe the parameters perturbed in the ensemble simulation instead of putting the PISM user’s manual reference. Which is the “SIA” parameter, for instance? Which is the basal sliding parameter considered? I think this would be much interesting for a broad audience of ice-sheet modellers than simply look at the parameter name and try to guess what it means.

A: As suggested, brief introduction of these parameters are given in the revision. For convenience, I copied these statements here.

‘The parameter till_effective_fraction_overburden parameter affects the effective pressure on the till. Lower till_effective_fraction_overburden value helps the ice to slide more easily. The parameter thickness_calving_threshold provide the thickness threshold for ice shelf calving. The temp_lapse_rate gives the lapse rate for vertical air temperature downscaling. The meltfactor_pik determines the efficiency of the heat exchange between ocean water and the grounded ice. Higher meltfactor_pik promote more ocean-ice heat exchange. The flow enhancement factor for the shallow ice approximation is given by the sia_e. More detailed information of these parameters can be found in PISM user's manual. ’

The following typos and grammar errors have been corrected accordingly.

Title: please, change it to “Impact of paleo climate on present and future evolution of the Greenland Ice Sheet”
Abstract: line 7, “… the climate of Greenland”.

Author summary: line 11, “of the GrIS is not only controlled...”
Line 9 (also further in the text): extent not extend

Line 38: change to “we simulate the evolution...”
Line 40 “which do not contain...”
Line 59: “It should be noted…”
Line 113: “we perform”
Line 162: “the 21 ka experiment features...”
Line 268: “model simulations reveal...”
Line 311: “the GrIS has undergone...”

References

Applegate, P. J., Kirchner, N., Stone, E. J., Keller, K., & Greve, R. (2012). An assessment of key model parametric uncertainties in projections of Greenland Ice Sheet behavior. The Cryosphere, 6(3), 589-606.
Bradley, S. L., Reerink, T. J., Van De Wal, R. S., & Helsen, M. M. (2018). Simulation of the Greenland Ice Sheet over two glacial–interglacial cycles: investigating a sub-ice-shelf melt parameterization and relative sea level forcing in an ice-sheet–ice-shelf model. Climate of the Past, 14(5), 619-635.
Buizert, C., Keisling, B. A., Box, J. E., He, F., Carlson, A. E., Sinclair, G., & DeConto, R. M. (2018). Greenland‐wide seasonal temperatures during the last deglaciation. Geophysical Research Letters, 45(4), 1905-1914.
Calov, R., Beyer, S., Greve, R., Beckmann, J., Willeit, M., Kleiner, T., ... & Ganopolski, A. (2018). Simulation of the future sea level contribution of Greenland with a new glacial system model. The Cryosphere, 12(10), 3097-3121.
Choi, Y., Morlighem, M., Rignot, E., Mouginot, J., & Wood, M. (2017). Modeling the response of Nioghalvfjerdsfjorden and Zachariae Isstrøm Glaciers, Greenland, to ocean forcing over the next century. Geophysical Research Letters, 44(21), 11-071.
Cowton, T. R., Sole, A. J., Nienow, P. W., Slater, D. A., & Christoffersen, P. (2018). Linear response of east Greenland’s tidewater glaciers to ocean/atmosphere warming. Proceedings of the National Academy of Sciences, 115(31), 7907-7912.
Fürst, J. J., Goelzer, H., & Huybrechts, P. (2015). Ice-dynamic projections of the Greenland ice sheet in response to atmospheric and oceanic warming. The Cryosphere, 9(3), 1039-1062.
Goelzer, H., Nowicki, S., Payne, A., Larour, E., Seroussi, H., Lipscomb, W. H., ... & van den Broeke, M. (2020). The future sea-level contribution of the Greenland ice sheet: a multi-model ensemble study of ISMIP6. The Cryosphere, 14(9), 3071-3096.

Rückamp, M., Greve, R., & Humbert, A. (2019). Comparative simulations of the evolution of the Greenland ice sheet under simplified Paris Agreement scenarios with the models SICOPOLIS and ISSM. Polar Science, 21, 14-25.

Tabone, I., Blasco, J., Robinson, A., Alvarez-Solas, J., & Montoya, M. (2018). The sensitivity of the Greenland Ice Sheet to glacial–interglacial oceanic forcing. Climate of the Past, 14(4), 455-472.
Jennings, A. E., Andrews, J. T., Cofaigh, C. Ó., Onge, G. S., Sheldon, C., Belt, S. T., ... & Hillaire-Marcel, C. (2017). Ocean forcing of Ice Sheet retreat in central west Greenland from LGM to the early Holocene. Earth and Planetary Science Letters, 472, 1-13.
Morlighem, M., Bondzio, J., Seroussi, H., Rignot, E., Larour, E., Humbert, A., & Rebuffi, S. (2016). Modeling of Store Gletscher's calving dynamics, West Greenland, in response to ocean thermal forcing. Geophysical Research Letters, 43(6), 2659-2666.
Rignot, E., Xu, Y., Menemenlis, D., Mouginot, J., Scheuchl, B., Li, X., ... & Fleurian, B. D. (2016). Modeling of ocean‐induced ice melt rates of five west Greenland glaciers over the past two decades. Geophysical Research Letters, 43(12), 6374-6382.
Syring, N., Lloyd, J. M., Stein, R., Fahl, K., Roberts, D. H., Callard, L., & O'Cofaigh, C. (2020). Holocene Interactions Between Glacier Retreat, Sea Ice Formation, and Atlantic Water Advection at the Inner Northeast Greenland Continental Shelf. Paleoceanography and Paleoclimatology, 35(11), e2020PA004019.
Slater, D. A., Felikson, D., Straneo, F., Goelzer, H., Little, C. M., Morlighem, M., ... & Nowicki, S. (2020). Twenty-first century ocean forcing of the Greenland ice sheet for modelling of sea level contribution. The Cryosphere, 14(3), 985-1008.
Yan, Q., Zhang, Z., Gao, Y., Wang, H., & Johannessen, O. M. (2013). Sensitivity of the modeled present‐day Greenland Ice Sheet to climatic forcing and spin‐up methods and its influence on future sea level projections. Journal of Geophysical Research: Earth Surface, 118(4), 2174-2189.

Reviewer #2: Review of “Impact of paleo climate on present and future evolution of Greenland Ice Sheet” by Tijn Berends

General comments

The authors present a revised version of a manuscript describing simulations of the Greenland ice sheet throughout the last glacial cycle and into the near future, using the ice-sheet model PISM forced with output of the AWI-ESM climate model. They find that, in agreement with the general consensus, changes in ice volume lag changes in climate by up to several millennia. Given that the Earth’s climate, particularly in Greenland, is thought to have been relatively warm during the early Holocene, and to have cooled to pre-industrial levels during the last few thousand years, this means that the Greenland ice sheet was likely not in equilibrium, but rather was still advancing when anthropogenic climate change commenced. The authors show that this likely delayed the onset of ice-sheet retreat until several decades after anthropogenic warming started, and is even now leading to slower ice-sheet retreat than would have been the case if the Holocene climate had been completely stable. These are important findings, as they highlight the need to account for the paleoclimatic history of the ice-sheet when projecting its future evolution.

In response to comments raised in the first review round, the authors significantly altered the manuscript. They included results from several additional experiments intended to investigate the sensitivity of their results to different model parameters, which support their conclusion that the observed lag in ice volume change with respect to climate is robust. They also altered the general story of the manuscript to focus more on the impact of (recent) paleoclimate change on near-future ice-sheet evolution, which has improved the readability of the manuscript. Lastly, they adequately addressed my major concern from the first review, which was unfortunately based on a misunderstanding on my part. I think the manuscript is now fit to be published after some minor revisions.

A: Thank you for your time to review our paper again. Your comments are very helpful.

Specific comments

Line 35: “Considering that the available studies focus on the evolution of the GrIS either in the past or into the future… ” This is phrased too strongly. Particularly the recent initMIP-GRL paper by Goelzer et al. (2018) presents a detailed study of the effect of model initialization (including paleo spin-up) on future projections. Mention this.

A: The first reviewer also raise the same comment. Please check the response to a previous comment. Goelzer et al. (2018) is referred in both introduction and discussion.

Line 43 (what happened to the line numbers here): “we select the Lingle and Clark bed deformation model…” I now understand the difference between your prescribed sea-level forcing and the dynamically calculated bed deformation. My previous criticism was based on a misunderstanding of this on my part, for which I apologise. I agree that the eustatic signal plus local bedrock deformation is probably sufficient for Greenland.

A: We did not clearly document this detail in the previous version.

Line 43 (idem): “A constant temperature lapse rate of 5 ◦C km−1 is adopted…” What is this based on? It seems a little low to me (between 6 – 8 K/km is common I think).

A: Indeed, this is a parameter which has large uncertainties. The tropospheric lapse rate γ is about 6.1 K/km in the Northern Hemisphere (NH) as a whole and over the ocean and about 6.2 K/km over the continents. The value of γ decreases from 6.5 K/km at low latitudes to 4.5 K/km at polar latitudes (ref to [. I. Mokhov](https://link.springer.com/article/10.1134%2FS0001433806040037#auth-I__I_-Mokhov) & [M. G. Akperov](https://link.springer.com/article/10.1134%2FS0001433806040037#auth-M__G_-Akperov) 2006:Tropospheric lapse rate and its relation to surface temperature from reanalysis data). Previous GrIS simulation used a value ranging from 4 to 8 K/km. We used a value of 5 and did another ensemble simulation with lapse rate of 6. It does not affect our conclusions.

Line 43 (idem): “The eigencalving and thickness calving parametrizations are used to determine the calving-front dynamics…” These are different approaches. Do you mean you use both of them together?

A: Yes, calving will take place when the ice front meet either of these two laws. This statement is added in the revision.

Line 57: “We tune the enhancement factor…” This requires more explanation. Do you do this in a steady-state set-up? Or do you tune it to give good results at the end of your LGC run? If so, do you also tune for LGM volume/extent in any way?

A: We do it only at the end of the paleo GrIS simulation (i.e, 125-0 ka). In the revision, we add a detailed statement for this point. We did not tune the LGM volume and extent. There is no common agreement on how much ice volume and extent was during the LGM.

‘…to get a reasonable ice volume (i.e., close to 7.4 m SLE) for the Pre-Industrial era (i.e., the end of the paleo GrIS simulation)’

Line 60: “…we do not use the elevation induced precipitation downscaling, because…[] …our climate model has a higher spatial resolution…” Is this spatial resolution smaller than the change in ice margin position? Particularly in the south-west, where the ice margin migrates onto the continental shelf during the LGM (presumably, actually a figure showing a few time-slices of your modelled ice-sheet geometry would be very nice!), you’d expect the high-precipitation area to migrate along with it. If you don’t correct for this in some way, this might introduce a bias in your mass balance.

A: You have pointed out an interesting point that most of the paleo ice sheet model does not take into account. To my knowledge, the available precipitation correction only applied on the vertical scale, i.e., when the elevation increase, precipitation should be decreased. However, in reality, precipitation pattern could also change, when the ice sheet retreat.

Unlike the Laurentide ice sheet, the GrIS margin do not have dramatic change during the last glacial cycle, therefore, the geometry induced precipitation pattern change is not that large either. Moreover, the different time slide climate simulations have already taken into account the ice geometry change. Interpolation between LGM and LIG precipitation pattern partly involve the geometry induced precipitation change. However, if we run the model into far future, when the GrIS melt away, the geometry induced precipitation pattern is expected to cause significant differences on the model results. In an offline coupled model, it is almost impossible to account this fact. This could be better resolved in a fully coupled climate-ice sheet model in the future.

In the revised discussion, we have addressed the shortcoming of the current simulation and the future direction of similar simulation between line 402-409.

Line 76: “…following the protocol of the Paleoclimate Modelling 76 Intercomparison Project phase 4…” Did AWI-ESM participate in PMIP4? How do your paleoclimate time slices compare?

A: Yes, AWI-ESM has participated the PIMP4 project. There are several papers published on the comparison of different model results within the PMIP4 activities, AWI-ESM produce similar climate pattern as the other models. We added a statement and referred the related PMIP4 papers in the revision, see line 135-139.

Line 94: “From 21ka to Pre-Industrial era, the AWI-ESM 94 time-slice simulations … are linearly 95 interpolated to derive a continuous climate forcing” Looking at Fig. 4A, this seems to introduce a warm bias during the deglaciation. Do you think this might affect your results?

A: Review #1 has similar comment on this point. Please check our response to the second comment from reviewer #1. Ice core is not the real observations. It has mixed the precipitation and temperature signals. A slight change of the climate forcing would change the modelled output, however, it will not affect the conclusion of this study. In the revised discussion, we have discussed the shortcoming of this study, i.e., glacial index, offline coupling, not realistic ocean forcing.

Table 1: I think you changed the SIA flow enhancement factor? Why not also the SSA factor?

A: Many model tuning effort do change both SIA and SSA. These model parameters have large uncertainties, according to the previous study and PISM manual. The purpose of carrying out ensemble simulations is to test whether different parameters lead to complete different results. Changing parameters is to introduce some noise into the ice sheet model. So, many parameters can be tested. There is no specific reason why we chose to only alter SIA enhancement.

Section 2 in general: maybe consider summarizing this. Your article seems to be aimed mostly at ice-sheet modellers such as myself. I don’t really know enough about the details of paleoclimate at specific points in time to know what to make of this paragraph. Really all I need to know is that your paleoclimate is good. If AWI-ESM participated in PMIP4/PMIP5 (while you refer to the PMIP protocol earlier, you don’t mention if AWI-ESM participated or not) then just mentioning that would already be enough.

A: We have a statement that ‘part of the above simulations participate in the fourth phase of the Paleoclimate Modelling Intercomparison Project (PMIP4)’. In the revision, we add another line to clarify this: ‘Model intercomparison indicates that AWI-ESM produces reasonable paleo climate patterns as compared with the other PMIP4 models (Kageyama et al. 2020, Kageyama et al. 2020, Shi et al. 2020, Brierley et al. 2020, Lohmann et al. 2020, Brown et al. 2020, comparison, Otto et al., 2021).’

Line 191: “…suggesting that the surface mass balance is the primary driver for the GrIS.” Given that at present, the GrIS loses most of its mass through glacier discharge (e.g. King et a., 2018; Fettweis et al., 2020) rather than runoff, I’d phrase this differently.

A: Sorry for the confusion. We want to address that after the Holocene, the oceanic forcing plays a minor role, while atmospheric forcing is the dominate driver for the GrIS. We have corrected the statement by changing ‘surface mass balance’ into ‘atmospheric forcing’.

Line 197: “…with an ice volume of 0.24 m SLE smaller than the Pre-Industrial volume.” I’d love to see a figure of this minimum extent.

A: Yes, the ice thickness, extent and surface elevation is given in the supplementary figures S3 and S4.

Line 232: “…In this simulation, the initial condition is obtained by continuing the paleo GrIS simulation for ten thousand years.” In my opinion, the difference between this simulation and the “default” run is your most important result; this illustrates very clearly why including a paleo-spin-up in future projections is so important. I’d mention this one already in the introduction, and consider rearranging the text of your results to present it accordingly (as in, you’ve done your default run and this one, plus a small ensemble of model parameter sensitivity runs for both experiments, so group them like that).

A: We revised our introduction. The model results are introduced on the basis of ensemble mean.

Line 268: “…reveals that the 268 GrIS may retreat…” Mind your grammar.

A: Corrected.

Line 281: “Our simulated magnitudes show discrepancies with the previous studies, likely because the simulations have not been constrained by any observation.” Elaborate on this. Which previous studies are these? What ice volumes did they find?

A: Detailed information is provided in the revision:’ Previous simulations (Simpson et al. 2009 and Lecavalier et al. 2014) constrained by observations suggest that during the last deglaciation, the GrIS may hold 4.6-5.1 m SLE more ice relative to present-day. Our simulated magnitudes show discrepancies with the previous studies, likely because the simulations have not been constrained by any observation. In addition, a simplified glacial index method could also contribute to such a discrepancy. ‘

Line 334: “Based on our results, we suggest that properly reproducing the ice velocity, or the rate of dynamic ice loss, is necessary to project the future evolution of the GrIS correctly. Thus, the paleo-spinup and assimilation approaches are proposed to be better methods to initialise the GrIS.” You’ve not done anything with data assimilation in your study, so this statement does not belong here. Whether or not a data-assimilated model will implicitly contain the information from the paleo-history of the ice-sheet is a question far beyond the scope of your study.

A: We agree. We have remove the ‘data assimilation’ statement.

Line 345: “At that time the greenhouse gases level was similar or even lower than today…” CO2 proxies that far back have uncertainties of over 50 ppmv in either direction, and data points for the Late Pliocene / Early Pleistocene cover pretty much everything from 250 to 450 ppmv (see e.g. Berends et al. 2021, Clim. Past.). Also, you’re now comparing centennial-scale sea-level rise to equilibrated ice volumes – which, as is pretty much the entire point of your study, are not at all the same.

A: Yes, our current equivalently CO2 (i.e., CO2+CH4+N2O) is on the order of 450 ppmv, which is similar or larger than the CO2 level around the Late Pliocene / Early Pleistocene. We have emphasized that the GrIS is sensitive to climate change, especially in the context of equilibrium response.

„At that time the greenhouse gases level was similar or even lower than today (Berends et al. 2020, Christ et al. 2021), hinting that the GrIS may be very sensitive to climate change, especially in the context of equilibrium response. “

Throughout the manuscript: mind the difference in spelling between the verb “to extend” and the noun “extent”.

A: Corrected.
