# Supplementary material for: Impact of paleoclimate on present and future evolution of the Greenland Ice Sheet
Source: PLoS One. 2022 Jan 20;17(1):e0259816. doi: 10.1371/journal.pone.0259816 (PMC8776332; doi:10.1371/journal.pone.0259816)
Supplement: Attachment — Submitted filename: PONE_response_letter.docx [file pone.0259816.s004.docx]

Response to Reviewers

Dear Editor,

Here is the point-to-point answer to the reviewer. All the line numbers refer to the track change version.

Regards,

Hu Yang

Reviewer #1: Yang et al. investigate the influence of the past climate of the Greenland Ice Sheet (GrIS) on its future projections of mass loss. By carrying out ice-sheet-earth-system models simulations from the last glacial period to the near future (2100 AD), they suggest that a classic long paleoclimatic spinup is needed to prevent an overestimation of predictions of Greenland ice loss.

I am glad the authors decided to redo the simulations to include the ocean as an additional forcing to the ice sheet model. I appreciate the authors' effort in this sense as I understand how this might be a considerable investment of resources, especially when dealing with paleoclimatic runs.

As the authors show in the revised manuscript, the inclusion of a spatially-uniform transient oceanic temperature forcing does not have a significant effect on the simulated GrIS evolution. I agree that this might be related to the simplistic approach adopted here. In fact, subshelf temperatures at depths lower than 100 m are likely more suitable to be considered, as closer to the ice shelf base. At those depths, the ocean water is decoupled from the surface air temperature, thus a transient oceanic temperature that follows the NGRIP time series is probably not a good representation of the paleoclimatic water temperature trend. An example can be found in southeast Greenland where warmer subsurface currents during the Younger Dryas, in clear contrast with the simultaneous atmospheric cooling, led to a significant coastal mass loss (Rainsley et al., 2018). Moreover, ocean temperatures around Greenland are strongly affected by local currents which, of course, cannot be captured if their spatial average is taken. However, since increased atmospheric temperature driven by insolation changes is likely the main responsible for the GrIS retreat during the Holocene (Vasskog et al., 2015), I would not expect that a more sophisticated approach regarding the ocean would drastically change the main results of this work, especially regarding the latest part of the Holocene. I therefore thank the authors for their effort made in satisfying my requests and I suggest the publication of the manuscript after minor revisions.

A: The shortcoming of our study in term of reproducing the regional ocean currents for sub-shelf melt is discussed in the revision between lines 383-388. For convenient, I also copy these lines here:

*‘Second, a spatially uniform ocean temperature is adopted to force the GrIS. This neglects the role of regional ocean current in driving the sub-shelf melt. In addition, our ocean temperature forcing follows the evolution of the NGRIP ice core. In reality, ocean temperature variations may decouple from the atmospheric temperature*

*variations as reconstructed from the Greenland ice core [91].’*

Specific comments

Abstract, line 8: please change to "maximum and minimum ice volume".

A: Modified.

Author summary
- line 3: please change to "it remains uncertain on how fast and how much the GrIS will contribute to it".

A: Modified

- line 8: I don't understand this sentence very well: "These results are consistent with evidence of the times of both past extremes in climates...". Please rephrase.

A: This sentence is rewrote as *‘These results are consistent with reconstruction evidence of the times of both past extremes in climates and the peak ice volume of the GrIS.‘*

- line 14: please change "growing" to "growth".

A: Modified

Main text:
Line 39: The short term response of ice sheet models to what? Please explain.

A: Modified to Line 39-41: ‘*Aschwanden et al. [32] found that the ice sheet model projected short-term evolution of ice sheet is strongly influenced by the initial condition of the simulation.’*

Line 64: This is imprecise as the PDD is an ablation scheme only. Please, correct.

A: Corrected to line 65-66: *’The Positive Degree Day (PDD) scheme is used to compute the ice ablation.‘*

Line 71: Please change to "at the ice front".

A: Corrected.

Line 75: Please change to "continental shelf of Greenland". This error repeats several times. Choose either "of Greenland" or "of the Greenland Ice Sheet".

A: Corrected.

Line 101: please change to "coupled with the".

A: Corrected.

Line 104: I think it is sufficient to write the acronym.

A: OK. Modified.

Line 126: please change to "the index to simulate".

A: Corrected.

Line 139: Which "situation"? Please consider changing it to "climate snapshots available before 21 ka" or something alike.

A: Adopted.

Line 143-145. I would be careful with this sentence. The parametrisation you are using (Beckmann & Goose, 2008) is built for the ice shelf base as no lateral fracturing is considered. Grounding lines in Greenland are usually deeper in the water column (Wilson et al., 2017), which contrasts your choice of considering an oceanic temperature at 100 m. I would add a brief sentence in the discussion regarding this and the possible influence that this might have on your results (see my general comments).

A: This sentence was removed. Moreover, the discussion of impact of regional ocean current on sub-shelf melt is presented in the revised discussion, see the answer to the very first comment and lines 383-387.

Line 149: I guess you wanted to put "PISM" instead of "PIK".

A: I replace the sentence by more detailed way as line 156-157: ‘*The ice shelf–ocean interaction parameterization introduced by Beckmann and Goosse …’*

Line 162: I would not call them "ensemble simulations" as they are rather sensitivity runs. This is misleading as it may be understood as five sets of simulations, while here we have only five runs. I would prefer to read it as "five simulations" or "five sensitivity tests" but I leave it to the authors' discretion.

A: We prefer to use ‘five simulations‘ and modified accordingly.

Line 176: "one more ensemble simulation" as above.

A: ‘ensemble’ is removed.

Line 195: what do you mean with "there are spatially heterogeneous anomalies at 9 ka"? Do you want to point out that the west Greenland is cooler than the Pre-industrial level, while the rest of the ice sheet is warmer? Please explain.

A: This sentence is modified to Line 206-209: ‘*There are spatially heterogeneous anomalies at 9 ka with west Greenland cooler, while the rest of the ice sheet warmer than the Pre-industrial*

*pattern.’*

Line 197: "This implies a long term cooling trend ...". The sentence before does not allow to get to this conclusion. Please, rephrase.

A: The previous sentence was replaced by Line 212-214: ‘*Our climate model simulations well capture the relatively warm Holocene climate as revealed by multiple proxy records.’*

Line 214: Figure S1 maybe?

A: Corrected

Line 219: Again, Fig. 6 is likely wrong.

A: Corrected.

Line 220: Was the acronym LGM presented before in the text?

A: It was introduced in the revision.

Line 228: "is relatively heterogeneous" maybe?

A: Corrected.

Line 241: Please change to "the minimum ice volume".

A: Corrected.

Line 249: Please check the figure numbers.

A: Corrected.

Line 282-284: Figure 2?

A: Corrected.

Line 298-300: I don't understand why the northeast has a higher volume if the precipitation rate is lower. Could you explain it better?

A: This sentence was rewritten. Line 313-316: *‘Increased ice volume is primarily found over the northeast (Fig 8a), where the precipitation rate is relatively low. Because the region with low precipitation rate needs more time to adjust (or gain mass) to a cooling climate.’*

Line 310: is this ice surface temperature or is it averaged over the ice column?

A: It is the average temperature over the entire Greenland ice sheet. Detailed explanation is given in the revised figure caption.

Line 312: Would you mean "the ice temperature of the GrIS is still increasing ..."?

A: Yes, corrected.

Line 406-415: This paragraph needs some rephrasing in my view. Contributing to 33mm to sea level rise by 2100 does not imply that the GrIS is rather little sensitive to climate change. This is a subjective point of view.

A: This sentence is modified as Line 435-437: ‘*This seems to indicate that the contribution of melting GrIS to sea level rise is only on the order of tens of centimetre, in the worst case.’*

References:
Beckmann, A., & Goosse, H. (2003). A parameterization of ice shelf–ocean interaction for climate models. Ocean modelling, 5(2), 157-170.
Rainsley, E., Menviel, L., Fogwill, C. J., Turney, C. S., Hughes, A. L., & Rood, D. H. (2018). Greenland ice mass loss during the Younger Dryas driven by Atlantic Meridional Overturning Circulation feedbacks. Scientific reports, 8(1), 1-9.
Vasskog, K., Langebroek, P. M., Andrews, J. T., Nilsen, J. E. Ø., & Nesje, A. (2015). The Greenland Ice Sheet during the last glacial cycle: Current ice loss and contribution to sea-level rise from a palaeoclimatic perspective. Earth-Science Reviews, 150, 45-67.
Wilson, N., Straneo, F., & Heimbach, P. (2017). Satellite-derived submarine melt rates and mass balance (2011–2015) for Greenland's largest remaining ice tongues. The Cryosphere, 11(6), 2773-2782.
